# Hydroxyapatite Dental Inserts for Tooth Restoration: Stress and Displacement Analysis

**DOI:** 10.3390/jfb16030075

**Published:** 2025-02-20

**Authors:** Maja Lezaja Zebic, Aleksandar Bodic, Djordje Veljovic, Tamara Matic, Jelena Carkic, Vladimir Milovanovic

**Affiliations:** 1 School of Dental Medicine, University of Belgrade, Nebojsina 35, 11000 Belgrade, Serbia; jelena.carkic@stomf.bg.ac.rs; 2 Faculty of Engineering, University of Kragujevac, Sestre Janjić, 34000 Kragujevac, Serbia; vladicka@kg.ac.rs; 3 Faculty of Technology and Metallurgy, University of Belgrade, Karnegijeva 4, 11000 Belgrade, Serbia; djveljovic@tmf.bg.ac.rs (D.V.); tmatic@tmf.bg.ac.rs (T.M.)

**Keywords:** tooth restoration, finite element analysis, hydroxyapatite insert, stress and displacement of the restoration, restoration failure

## Abstract

Hydroxyapatite (HAP) inserts minimize restoration contraction by constituting a major part of the restoration; however, their effect on the relaxation of tooth tissues has not been previously tested. Finite element analysis was employed to estimate stress and displacement when HAP inserts with a thickness of 1.7 mm or 4.7 mm and a diameter of 4.7 mm were used to substitute for dentin. The volumetric contraction of the composite during polymerization, simulated through steady-state heat transfer analysis, yielded a contraction rate of 3.7%. Descriptive statistics revealed that the incorporation of HAP inserts reduced the displacement of dentin, enamel, and restoration caused by contraction by 44.4% to 66.7%, while maximal stress was reduced by 8.1% to 52%. Subsequent loading on the occlusal tooth surface showed that displacement values decreased by 12.1% to 33.3%, while maximum von Mises stress in enamel decreased by 32.8% to 40.6% with the use of HAP inserts. Although the maximum stress values in dentin were not significantly decreased (3% to 8.8%), the stress located at the bottom of the cavity was notably reduced, particularly in deep cavities at root canal entrances. The use of HAP inserts in restorative dentistry provides benefits for the preservation of prepared teeth, especially in preventing irreparable vertical root fractures of endodontically treated teeth.

## 1. Introduction

Tooth cavity preparation is an inevitable part of caries management that results in weakened tooth structure, leading to more frequent tooth fractures compared to healthy teeth. The restorative procedure and the materials used for the restoration are the only factors that can be adjusted to minimize this occurrence [1]. Numerous attempts to reduce polymerization contraction have been based on modifying materials that continue to polymerize in the cavity, and therefore contract [2,3,4]. The approach of using inserts, which do not change the dimensions of the cavity, aims to significantly reduce the amount of material that polymerizes in the cavity. The concept of mitigating the adverse effects of polymerization shrinkage in dental composites through the use of dental inserts—i.e., prefabricated components similar to inlays, but without individualized shapes—dates back to 1989 [5]. The first inserts, called megafillers, were used for restorations [6,7], and later, ceramic inserts of various sizes made from bio-inert ceramics like IPS Empress, β-quartz glass ceramic, and leucite-reinforced ceramic were introduced [8,9]. Conversely, hydroxyapatite (HAP), a bioactive, biocompatible, and osteoconductive material with desirable mechanical and thermal properties, has been widely used for biomedical applications in the repair and replacement of injured or damaged teeth and surrounding bones [10]. HAP ceramics, used as dental inserts for dentin restoration, were first tested in 2015 [11], and have since been further examined in several studies [12,13]. These investigations include the evaluation of the shear bond strength of HAP inserts with various restorative dental materials. A recent study by Matic et al. [13] was the first to examine the effect of HAP inserts on the ultimate fracture resistance of restored teeth compared to teeth restored with composite alone. However, the variability of the human teeth used in the study made it challenging to determine the true impact of the HAP inserts. To address this limitation, Finite Element Analysis (FEA) can be employed to evaluate stress generation and displacement occurrence, including their precise locations within such restorations. This approach avoids invasive procedures and ethical concerns while providing complete repeatability, easy and clear interpretation of results, visualization, and precise force application [14,15].

Finite Element Analysis (FEA) is the most common numerical method for efficiently analyzing biomechanical problems [16]. In the field of dentistry, this method is often used in implantology [17,18], oral radiology [19,20], orthodontics [21], prosthodontics [22], and restorative dentistry [23]. The FEA method, due to its versatility and ability to analyze biomedical problems—especially those that are difficult to assess in vivo and are challenging due to structural complexity—is not only a very useful tool but also saves time and resources for conducting experiments.

The main issue with using dental composites in tooth restoration is their contraction during polymerization. Modern composite resins undergo volumetric contraction ranging from 2.6% to 4.8% [24]. This contraction competes with bond strength and may lead to marginal disintegration. While modern dentin bonding agents exhibit bond strengths to dentin exceeding 20 MPa [25], the contraction stress generated during polymerization (13–17 MPa) approaches these values. When bond strength is not exceeded, the contraction generates stress and displaces tooth tissue, posing a risk of marginal failure when combined with masticatory loads. Additionally, temperature fluctuations associated with food and beverage consumption create additional stresses in the bonding area. This is due to dental composites having a thermal expansion coefficient in the range of 25–60 ppm°C^−1^, which is significantly higher than that of enamel (11.4 ppm°C^−1^) and dentin (8 ppm°C^−1^) [26]. In contrast, HAP-based ceramics exhibit a thermal expansion coefficient of about 13.3 ppm°C^−1^, closely aligning with the natural tooth structures due to their chemical similarity to the inorganic part of dentin and enamel [27].

In our previous studies, HAP inserts were produced as 2 mm thick disks. However, the beneficial effects of these inserts could potentially be greatly emphasized by increasing their thickness. A logical first step toward achieving this would be to simulate this situation via mathematical modeling and FEA. This study is the first to use FEA as a tool for analyzing dental inserts, especially HAP-based inserts.

Inserts, including HAP inserts, are designed for direct restorative procedures and serve as an alternative to composite restorations. Their primary purpose is to reduce the amount of material that undergoes polymerization within the cavity. To evaluate their effectiveness, it was necessary to measure the stress reduction caused by polymerization contraction through simulation. Polymerization-induced contraction stress remains within the tooth and combines with additional stresses during mastication. This highlights the advantage of performing multi-step analyses in finite element analysis (FEA), as it accounts for both types of stresses, providing a more accurate simulation of clinical conditions.

The aim of the study was to test five tooth models: a healthy tooth, teeth with medium deep cavities restored with or without hydroxyapatite (HAP) inserts, and very deep cavities restored with or without HAP inserts. The cavity was designed in the shape of a cylinder with variable height, simulating the form of a Class I cavity restoration. Models were loaded in two phases: firstly, contraction simulation, and subsequently, the occlusal surface loading. The maximum stress generated, maximum displacement occurrence, and stress distribution were analyzed.

The first hypothesis was that there would not be a difference in the stress generated in dental tissues and the restoration itself among teeth restored with composite alone or cavities restored using hydroxyapatite (HAP) inserts for dentin replacement, with composites serving as a cover for the insert in the enamel portion of the tooth. The second hypothesis was that there would not be a difference in the displacement values among the tested models.

## 2. Materials and Methods

**Figure 1:**
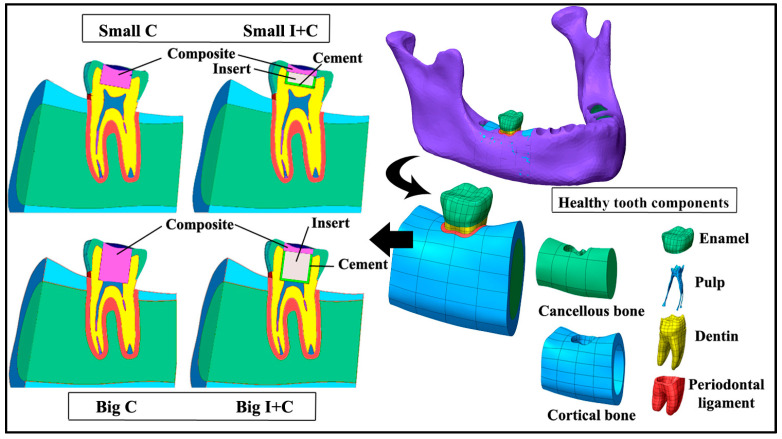
The right mandibular first molar was extracted from the jaw, along with adjacent bone, tested as a healthy tooth, and prepared for “Small” and “Big” cavity restorations. For the “C” groups, the restoration comprised only composite, while for the “I+C” groups, it included cement for insert cementation, the HAP insert itself, and the composite above the insert. A cross-section of the tested models, with marked components, is presented in the left portion of the figure.

For the purpose of numerical simulation, the geometry of the right mandibular first molar was reconstructed from the Open Mandible Source 3D scan [28] https://github.com/ArsoVukicevic/OpenMandible, accessed on 13 April 2021. The model included the morphological distribution of all dental tissues, so the dental tissues did not need to be subsequently separated, marked, or anything similar. To minimize the influence of boundary conditions on the numerical simulation, the geometry of the mandible in the immediate vicinity was also reconstructed. The 3D geometry of the tooth was generated utilizing non-uniform rational B-spline (NURBS) surfaces within patch boundaries established by polygonized points. The patch network was drawn manually to achieve higher quality surfaces. The deviation of the created surfaces from the mesh was less than 0.1 mm. Tooth height was 21 mm, the Vestibule-Oral dimension was 10 mm, and the mesial-distal dimension was 11 mm. Tooth geometry was further adapted for five scenarios: a healthy tooth and a tooth with two possible cavity depths—small cavity and big cavity—both restored with restorations comprising only composite (codes: Small C and Big C) in control groups. The diameter of the hole (cylinder preparation) was 5.5 mm, with two possible depths. In the experimental groups, the restorations were composed of a combination of insert, resin-based cement, and composite in the following way: HAP inserts with thicknesses of 1.7 or 4.7 mm and diameters of 4.7 mm for the small and big cavities, respectively (codes: Small I+C and Big I+C), cemented by resin-based cement as a bowl beneath and circumferentially around the insert with the thickness 0.5 mm, and covered with a top layer of composite above the insert in the enamel part of the tooth (Figure 1). The dimensions of the used lower first molar are provided in Figure 1.

For the model preparation, all materials were considered isotropic, homogeneous, linear, and elastic. The mechanical properties used for the models are summarized in Table 1, along with the sources of the data.

The models’ pre- and post-processing steps were performed using Femap software, version 21.2, while the calculations were carried out in Simcenter Nastran software. The finite element model included the following parts: mandible, periodontal ligament, dentine, pulp, enamel, composite, cement, and insert (the last two only in the experimental groups). All finite element models were created using tetrahedral finite elements with mid-side nodes. To achieve an optimal number of finite elements, different element sizes per volume were defined in various zones of the model. The average size of the finite elements in the tooth zone was 0.2 mm, increasing to 1 mm in the mandible zone. The Jacobian quality criterion was used as an indicator of the quality of the generated finite elements. This criterion compares the shape of a finite element with the “ideal shape” of an element of the same type. Valid elements have a Jacobian value between 0.0 and 1.0, with the first value representing an “ideally shaped” element. In the created finite element models, the Jacobian values of most finite elements were less than 0.7. In the dental pulp zone, a small number of finite elements (100 elements) had a Jacobian coefficient in the range of 0.7–0.85, where the shape of the elements is determined by the geometry and the total number of finite elements. The number of finite elements and nodes varies for different tooth models, with an average of approximately 500,000 finite elements and 740,000 nodes. The linear elastic static analysis (SOL 101) was performed using the Sparse Solver in Simcenter Nastran, which employs direct factorization and does not require iterative convergence criteria.

**Figure 2:**
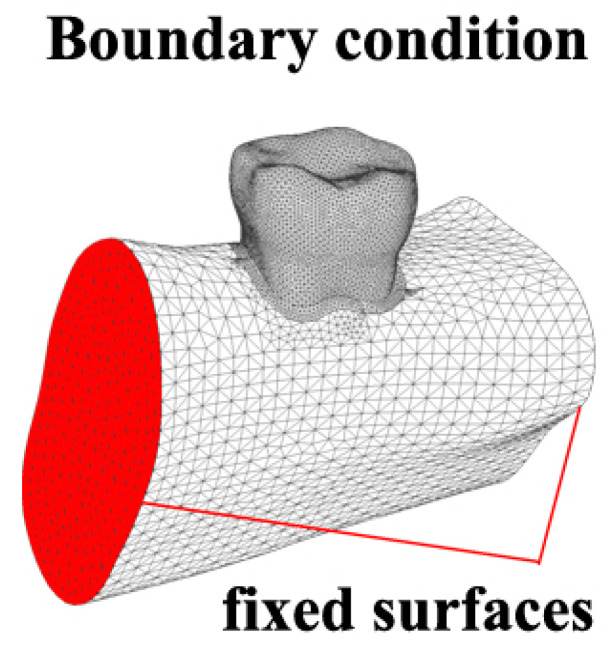
Boundary conditions.

Fixed boundary conditions were placed on the side surfaces of the mandible segment to minimize their influence on the model. The boundary conditions and finite element (FE) mesh are shown in Figure 2.

**Figure 3:**
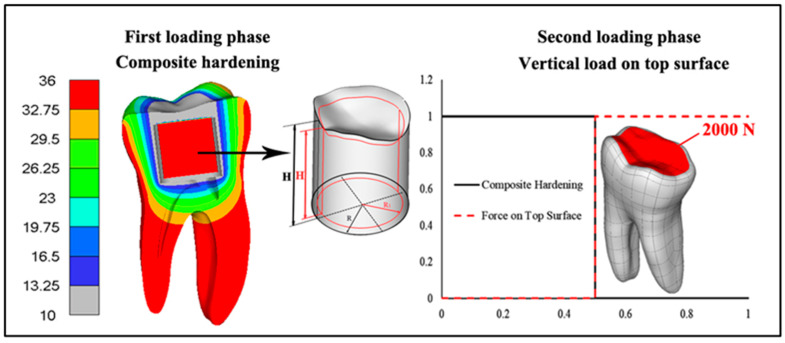
First and second loading phases were implemented in the simulation. The first phase involves simulating the composite polymerization contraction with the model to calculate the percentage of contraction. A tooth cross-section is presented, demonstrating the temperature used for contraction provocation. The second loading phase involves the contraction simulation followed by a load of 2000 N continuously distributed over the occlusal surface.

The load assigned to the model was a thermal load to cause the contraction of polymerizing materials (composite and cement for insert cementation), along with an extreme force on the entire occlusal surface of all models (2 kN) (Figure 3). Our recent research showed that tooth fracture resistance for teeth restored with or without HAP inserts was about 3–3.2 kN [13]. Therefore, we used a force value of 2 kN, which has also been used by other researchers [32,33].

The effect of polymerization contraction was achieved by applying a temperature field obtained from steady-state thermal analysis. Steady-state thermal analysis was performed under the following conditions: a temperature of 10 °C was set on all nodes of the composite and cement, while a temperature of 36 °C was defined on the nodes located on the external surfaces of the model and all nodes on the insert for the experimental groups (Figure 3). Since the volume of the restoration could be approximated as a cylinder, it is possible to compare the cylinder volume before and after the effect of the temperature simulation, considering displacement in all three directions and calculating new dimensions. The initial and reduced volumes can thus be determined, allowing for the calculation of the percentage of contraction (Figure 3).

After the tooth contraction was achieved due to the influence of the temperature field, the tooth was further loaded in the second loading phase with occlusal surface force (Figure 3, right side). The loading of the model by phases was regulated by the loading functions shown in Figure 3.

## 3. Results

**Figure 4:**
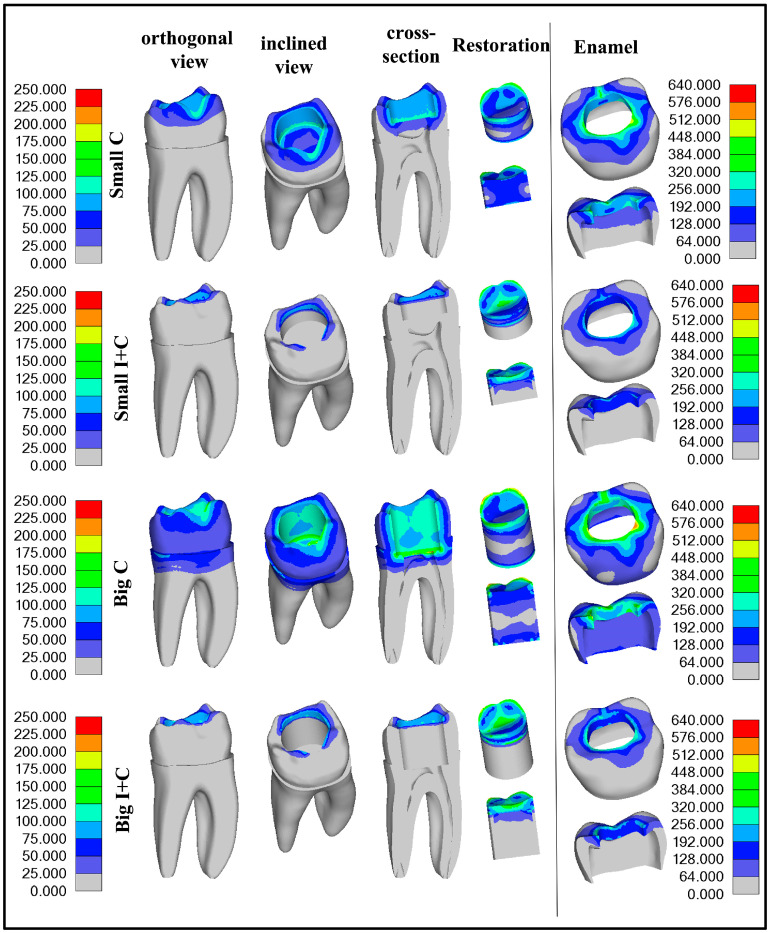
Graphical presentations of stress detected in all model components for the control (Small C and Big C) and experimental (Small I+C and Big I+C) first loading phase, which simulated polymerization contraction alone are provided.

**Figure 5:**
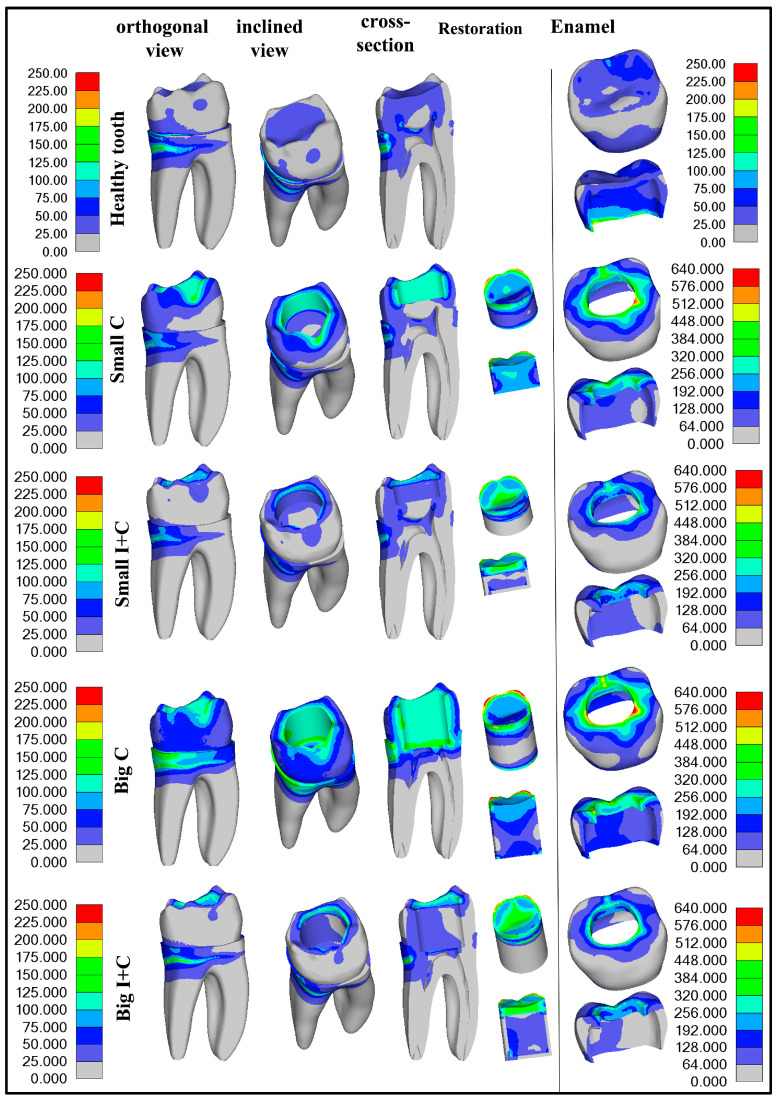
Von Mises stress distribution in healthy teeth after load application and for other groups after multistep analysis is provided. The legend value bar on the left side is common for dentin and the restoration, while the legend value bar on the right side is specific to enamel.

**Figure 6:**
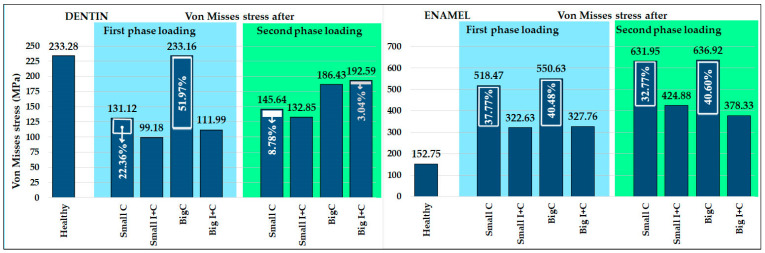
Maximal detected values of von Misses stress in dentin and enamel, for both loading phases with decrease percentages shown.

Results from the first loading phase, which simulated polymerization contraction alone, and the second loading phase, which included further load application, are presented graphically in Figure 4 and Figure 5, respectively. For better understanding of stress distribution, the same legend value bar was used for all models for dentin and restoration segments (0–240 MPa) and for enamel (0–640 MPa). The models were also set in identical views to provide visual comparability. The components of the models were separated based on the area into dentin, enamel, and restoration. Maximum detected values are presented in Figure 6, with decrease percentages shown within the bar of the control groups (Small C and Big C) for both loading phases. Maximum stress generated after contraction simulation is presented in Table 2. Maximum stress generated after applying occlusal load to healthy teeth are presented in Table 3. In Table 4, the results for the cavity groups are shown, showing the maximal stress values after both loads applied sequentially. Differences in the obtained maximum values among the control and experimental groups are presented as percentages (%) of change to facilitate the interpretation of the results. Descriptive statistical values are shown in Table 5. All displacement results for both simulation phases are presented comparatively in Table 6. 

### 3.1. Polymerization Contraction Results—First Loading Phase

The polymerization contraction achieved with temperature simulation was 3.7%. Results of maximum Von Mises stress caused by polymerization contraction are presented in Table 2 and Figure 6, while the distribution of the stresses is illustrated in Figure 4. The highest stress was observed in the enamel for all groups, and with an increase in cavity size, the stress values in all components slightly increased, as expected. The application of HAP-based inserts reduced the stress in all components: enamel, dentin, and restoration (Table 2 and Figure 4 and Figure 6). The most prominent reduction of Von Mises stress was found in dentin for the big cavity group, with stress values more than halved (51.97% decrease) due to the usage of HAP inserts. The reduction of stress was also pronounced for enamel in both small and big cavities (37.77% and 40.48%, respectively). Since the Von Mises stress values detected in the restoration represent the maximum values for both composite and insert, the stress reduction observed in the groups with inserts was moderate (8.07–11.67%). In insert-containing groups (Small I+C and Big I+C), the maximum stress detected was located in the composite layer, while deeper parts of the restoration generated less stress, which is expected as HAP ceramics are not affected by polymerization contraction. Consequently, dental tissues also showed higher stress in the superficial part of the experimental groups, which was not the case for the control groups, where the majority of the stress was located at the cavity bottom.

**Table 2:** Maximum Von Mises stress detected after polymerization contraction simulation analysis in MPa.

	Small C	Small I+C	Percent of Decrease	Big C	Big I+C	Percent of Decrease
Enamel	518.47	322.63	37.77%	550.63	327.76	40.48%
Dentin	131.12	99.18	22.36%	233.16	111.99	51.97%
Restoration	194.17	178.50	8.07%	205.68	181.67	11.67%

### 3.2. Healthy Tooth Results

A healthy tooth was subjected only to load application, as the temperature simulation had no target material in this case. The results of maximum stress and displacement of the healthy tooth after loading are presented in Table 3. The maximum stress of the healthy tooth was located in the distal root, below the enamel. The enamel–dentin junction at the tooth neck is a common site for maximum stress detection, which in this case was further influenced by the tooth’s inclination in the jaw and its morphology. The graphical presentation of healthy tooth results is combined with the multistep analysis (second load phase) results in Figure 5, and its maximum value is also presented in Figure 6.

**Table 3:** Maximum Von Mises stress and displacement after load applied on the healthy tooth.

	Max Von Mises Stress (MPa)	Max Displacement (mm)
Enamel	152.75	0.03
Dentin	233.28	0.03

### 3.3. Numerical Analysis Results After Second Loading Phase

Results of maximum Von Mises stress values caused by simulated polymerization contraction and subsequent load application on the top surface (Figure 3) are presented in Table 4 and Figure 6. Figure 5 graphically illustrates the stress distribution within the models. The legend value bar for the enamel of the healthy tooth differs from the bar of teeth with cavities, as it showed lower values, aligning more closely with those of dentin.

**Table 4:** Maximum Von Mises values (MPa) caused by polymerization contraction followed by the load simulation–multistep analysis results.

	Small C	Small I+C	Percent of Decrease	Big C	Big I+C	Percent of Decrease
Enamel	631.95	424.88	32.77%	636.92	378.33	40.60%
Dentin	145.64	132.85	8.78%	186.43	192.59	+3.04%
Restoration	243.18	224.16	7.82%	249.52	218.23	12.54%

The application of occlusal loading following the polymerization contraction simulation resulted in about a four-fold increase in stress levels in the enamel compared to a healthy tooth subjected only to the occlusal load. This highlights the fragility of a restored tooth due to compromised tooth integrity. Additionally, in the models with cavities, the stress is increased due to the polymerization contraction that precedes the loading. As in the simulation with polymerization only, and the highest stress values were obtained in the enamel for all groups, with a substantial decrease in the teeth restored with HAP inserts (32.77% and 40.60%), which was more pronounced in the larger cavity (40.60%). Stress reduction in enamel with the use of inserts is very important, as it is the first line of defense exposed to the oral environment, and overall stress in this zone could result in marginal restoration failure and bacteria penetration toward dentin.

Maximum stress generated in dentin was found to be less in all teeth with cavities than in the maximum stress of a healthy tooth (Figure 6). The use of HAP inserts only slightly influenced maximum stress detected in dentin; however, the stress distribution following the second loading phase differed greatly between the control and experimental groups (Figure 6). In the control groups, especially for the big cavity group (Big C), the main stress was located at the preparation bottom, directed toward the endodontic space, threatening to cause a vertical root fracture (Figure 5). In the experimental group (Big I+C), the maximum stress was located at the same spot as in the case of a healthy tooth—the distal root—with a relaxed cavity bottom, indicating that HAP inserts play a role in protecting the endodontic space from overall load, thereby lowering the risk for vertical root fracture. Figure 5 clearly demonstrates how the presence of HAP inserts reduces stress in the dentin, thereby providing protection to this crucial part of the tooth structure.

The stress generated in the restoration after the second loading phase was slightly lowered by 7.82% and 12.54% for small and big cavities, respectively, for the restorations comprising HAP inserts. In these groups (Small I+C and Big I+C), the maximum stress was concentrated in the composite layer, which forms the superficial layer of the restoration; thus, a more pronounced stress decrease was not anticipated. However, the deeper parts of the restoration exhibited reduced stress levels in the HAP insert groups, as illustrated in Figure 5.

A diagram with maximum detected values of Von Mises stress for both loading phases, for dentin and enamel, is presented in Figure 6, and descriptive statistical results for values obtained in each element of the tested models are presented in Table 5.

**Table 5:** Descriptive statistics for Von Mises stress values after second loading phase for all elements.

		Average	STDEV	1st Quart.	Median	3rd Quart.	Max.
Enamel	Small C	98.38	76.01	30.24	75.36	94.83	660.20
	Small I+C	68.36	49.48	24.41	55.85	68.92	455.84
	% reduction						
	Big C	113.70	69.16	45.08	100.10	119.07	636.92
	Small I+C	55.77	40.51	20.46	45.61	55.21	378.33
	% reduction						
	Healthy tooth	35.99	18.10	14.74	34.52	40.39	233.28
Dentin	Small C	28.45	28.48	2.58	21.13	29.05	180.61
	Small I+C	21.51	18.51	2.49	18.07	24.14	187.32
	% reduction						
	Big C	33.35	35.53	1.91	20.02	40.11	186.43
	Small I+C	16.89	15.87	1.61	13.81	18.96	192.59
	% reduction						
	Healthy tooth	18.79	15.86	2.12	16.21	21.76	152.75

### 3.4. Displacement Results

Results of the obtained displacement values for all models and both loading phases (polymerization only and multi-step analysis) are shown in Table 6. The displacement in the restoration greatly increased after the application of the load in the second simulation phase, with values similar to those obtained after loading the healthy tooth (0.03 mm).

Displacement values in the restoration following only the polymerization simulation were drastically decreased with the use of HAP inserts (44.44–58.33%), as well as in the enamel (43.75–55%) and in dentin (50–66.66%), indicating the important role of inserts during the polymerization contraction. Additionally, displacement values in the restoration after the second loading phase were also decreased for all three components explored—restoration, dentin, and enamel. This reduction was most pronounced in restorations, decreasing displacement in the groups containing inserts by 17.95% and 33.33% for small and big cavities, respectively. The reduction of stress was similarly pronounced for stress generated in dentin (14.29% and 30% for small and big cavity groups, respectively), and slightly less in enamel (12.12–16.48% for small and big cavity groups, respectively).

**Table 6:** Maximum displacement values (mm) caused by polymerization contraction or by polymerization contraction followed by the load simulation (multistep analysis).

		Small C	Small I+C	Percent of Decrease	Big C	Big I+C	Percent of Decrease
Polymerization only	Enamel	0.016	0.009	43.75%	0.020	0.009	55%
	Dentin	0.010	0.005	50%	0.015	0.005	66.66%
	Restoration	0.018	0.010	44.44%	0.024	0.010	58.33%
Multistep analysis	Enamel	0.033	0.029	12.12%	0.034	0.025	16.48%
	Dentin	0.028	0.024	14.29%	0.030	0.021	30%
	Restoration	0.039	0.032	17.95%	0.042	0.028	33.33%

## 4. Discussion

The first hypothesis was rejected, as the experimental groups showed different results in stress generated in tooth tissues compared to the control groups. Stresses generated in the restoration were also decreased in the experimental groups. The second hypothesis was also rejected, as displacement in the model components after polymerization was drastically decreased for experimental groups.

Testing the mechanical properties of biomaterials and human tissues in vivo represents a serious challenge [15]. Mathematical simulations have various advantages for biomedical materials analysis: they provide results that are comparable to studies conducted on real models, allow for repeatable and accurate testing, and eliminate ethical concerns [15]. In comparative studies, such as this one, all variations can be introduced within the same model, thus reflecting only true differences caused by the modified factor.

Hydroxyapatite-based dental inserts are prefabricated sintered bioceramic discs made by powder compressing and subsequent sintering. These inserts can be produced in geometrically uniform shapes, such as cylinders of different heights. HAP inserts are intended for use in direct restorative procedures, which is why they were compared with composite restoration in this study rather than with ceramic restorations. The cavity design, considering this indication, resembled a Class I cavity but with geometrically regular cylindrical contours. The terms “small” and “big” cavities refer to different cavity depths, while the cavity diameter remains constant. A small cavity corresponds to a restoration of a vital tooth with an intact pulp chamber roof, whereas a big cavity represents a devitalized tooth with the cavity floor extending to the root canal entrances. HAP inserts have previously been shown to effectively bond with commercially available dental materials and reduce polymerization contraction by minimizing the use of dental composite [12]. Although the application of HAP inserts was recently confirmed to have no negative effect on the overall fracture resistance of restored human molars compared to teeth restored with a resin-based dental composite [13], additional studies are needed to clarify the influence of HAP inserts on tooth structure. Unlike porous hydroxyapatite structures designed to interact with cells and bone tissue, HAP inserts are compressed and sintered hydroxyapatite ceramics with high stiffness (100 GPa). Thus, they were modeled as solid. To avoid biasing the comparison in favor of the insert, the composite material is also modeled as solid, despite this not reflecting its actual behavior in real clinical practice.

In the present study, finite element analysis (FEA) was employed to eliminate the variability of human teeth properties, as well as to separately examine the effects caused by polymerization contraction and occlusal surface loading. Previous studies have compared stress distribution patterns between direct and indirect resin restorations, reporting similar results between these groups; however, polymerization contraction was not considered in that analysis [34]. In this study, polymerization contraction was simulated prior to the ultimate load application, providing a more accurate representation of clinical conditions.

In the second loading phase, an ultimate load of 2000 N was uniformly applied over the entire occlusal surface, in accordance with the maximum force used in previous studies [32,33]. This load greatly exceeds masticatory forces, as the reported maximum bite force for vital and devitalized teeth after pulp removal is 207.93 N and 226.6 N, respectively [35]. While the forces applied are much higher than those encountered during normal chewing, the objective was to conduct a preliminary investigation into the effects of HAP inserts on overall tooth integrity under extreme conditions, in order to assess their maximum potential benefits.

Since this is the first study to test the effects of ceramic inserts on the stress distribution and displacement in the tooth structure, the results obtained can only be compared to ceramic prosthodontic restorations or composite restoration. It has been previously demonstrated that all-ceramic inlay and onlay materials generally transfer less stress to dental structures due to their stiffness [36], which is consistent with the results obtained in the present study.

HAP inserts offer distinct advantages over ceramic inlays and onlays, as they are easier to apply in clinical practice and do not require the production of custom ceramic shapes. Regarding cavity design, onlay design was found to be more effective in protecting tooth structures compared to inlays [36]. Inlays generate approximately 4.4% higher stress than onlays, making the onlay design preferable for cavity preparation [37].

However, such cavity design was not considered in the present study, which focused on everyday dental practice, where direct composite restorations are used. Therefore, the results of the insert group were compared to conventional composite restorations. Additionally, the application of inserts in direct tooth restoration requires the use of a composite topcoat, which makes such restoration distinct from ceramic inlays and onlays. Regarding dental composites, they produce greater stress compared to ceramics due to their significantly lower elastic moduli [36]. The study by Ouldyerou et al. compared amalgam, composite (with various Young’s modulus values), and glass ionomer restorations, concluding that composite restorations were the most favorable. They found that increasing the Young’s modulus of restorative composite material reduced stresses generated in enamel and dentin [38]. The Young’s modulus of the composite resin used in the present study falls within the middle range of the values tested in their study. Bio-inert dental ceramics exhibit stiffness ranging from 50 GPa in feldspathic porcelain to 250 GPa in glass-infused alumina or zirconia [39]. The HAP ceramics tested in the present study possess a Young’s modulus of about 100 GPa [21,29], placing them in the mid-range compared to other ceramics. This indicates greater brittleness compared to composites, but also higher load resistance [20]. Moreover, this parameter can be tuned by controlling the sintering process of HAP, allowing for the design of optimal grain and pore size, phase composition, and microporosity [40].

Whereas healthy teeth may break due to traumatic factors [3], endodontically treated teeth are more fragile than vital teeth and can fracture because of typical functional factors [41]. Tooth fracture is the third most common cause of tooth loss [42]. Krell and Rivera found that almost 10% of patients referred for endodontic evaluation and treatment over a 6-year period had a cracked tooth [43]. The main reason for such fractures is the loss of substance during caries treatment and the preparation of endodontic access cavities, especially if extended access cavities are prepared [44]. Removing the marginal walls, particularly in occlusal areas, during preparation negatively affects the fracture resistance of endodontically treated teeth [4]. Dehydration, collagen cruciate ligament loss, and dentin loss after endodontic treatment also negatively impact fracture resistance [45]. Vertical root fractures are primarily caused by uneven stress distribution around the middle part of the root canal [46]. Until now, a potential approach to mitigate these effects has been the use of base materials, such as resin-modified glass ionomer or fluid composite resin, placed beneath composite restorations. These base materials are reported to absorb stress; thus, they could be beneficial, although their effectiveness remains controversial [6,7,8]. HAP inserts tested in the present study demonstrated a significant role in reducing stress in the dentin zones at the entrance to the root canals of teeth. The results suggest that HAP inserts may offer great advantages in restoring endodontically treated teeth, particularly by protecting the remaining tooth tissue, especially in the deeper dentin zones. However, these findings should be further evaluated under dynamic loading conditions with clinically relevant loads. Additionally, the study by Ausiello et al. [47] showed that base material beneath bulk-fill composites can exacerbate stress concentration, while block composites, similar to the insert approach, may better mitigate stress.

The results of this study indicate that the application of HAP inserts reduced maximum Von Mises stress values caused by polymerization contraction in the tooth structure and restoration, with a more prominent effect on the tooth structure: 22.36–51.97% in dentin and 37.77–40.48% in enamel (Table 2). The positive impact of HAP inserts was especially visible in the larger cavities after the polymerization contraction simulation, with a 51.97% stress reduction in dentin. When the ultimate load was applied, the stress in enamel greatly increased; however, the application of HAP inserts reduced stress in enamel by 32.77% in small cavities and 40.60% in large cavities. These findings should be further evaluated, including potential flaws and defects in both the composite material and HAP inserts, using Taguchi’s robust parameter design to determine model sensitivity to input parameters. Further, the Weibull function could be combined with the FEA results to predict long-term failure probability [48], as probabilistic analysis allows for determining the distribution of a response variable based on the distributions of the input variables [49].

In addition to the maximum stress values, the distribution of stress is equally significant for preserving the integrity of restored teeth. In the control group, the primary stress was concentrated at the bottom of the preparation, directed toward the endodontic space, posing a risk of vertical root fracture. In contrast, the application of HAP inserts showed maximum stress located similarly to that in a healthy tooth—at the distal root—while the cavity bottom remained under less stress, as seen in Figure 5. These findings suggest that HAP inserts have a shielding effect on the endodontic space, thereby reducing the likelihood of vertical root fracture.

Partially substituting the tooth structure with a HAP insert not only reduced Von Mises stresses but also significantly decreased the displacement values caused by polymerization shrinkage across all tooth components, thereby minimizing the risk of gap formation. This effect was more pronounced when only polymerization simulation was applied. The reduction of displacement was less prominent after the subsequent load application, likely due to the high forces applied, but it was still notable, particularly in larger cavities.

Limitations of the present study, which are expected to be addressed in future research, include the idealized fitting of insert geometry to cavity geometry with a geometrically regular shape, the clinically irrelevant static load applied, and the modeling of elements as solid, without pores, gaps, or anisotropy and without rounded edge surfaces. Future studies should also investigate other cavity designs restored with HAP inserts, provide quantitative comparisons with existing data regarding both composite and ceramic restorations, and examine the effect of HAP inserts’ brittle nature on clinical handling and behavior in a clinical environment. However, it is beneficial that the properties of HAP inserts could be tailored by optimizing sintering parameters if the need arises.

## 5. Conclusions

The simulation of polymerization contraction was found to be responsible for the majority of the stress generated during the numerical analysis. The displacement of tooth tissues during the polymerization of the restoration was decreased by up to 66.7% with the use of HAP inserts. The maximum Von Mises stress in the enamel after the second loading phase was decreased by up to 40.60%. More importantly, in deep cavities, the use of the insert protected the dentin at the bottom of the cavity, near the root canal entrances, thus potentially contributing to the prevention of unrepairable vertical root fractures.

With the study’s limitations in mind, the obtained results suggest that HAP inserts should be further evaluated as possible contributors to the durability and stability of devitalized teeth by reducing stress and minimizing potential gap formation, especially in larger cavities. Longitudinal clinical studies are needed for further investigation. The results obtained suggest that HAP inserts should be further evaluated as contributors to tooth stability.

## Figures and Tables

**Table 1:** Mechanical properties of the considered component for the models.

Material	Young’s Modulus (MPa)	Poison’s Ratio	Source Reference
Pulp	6.8	0.45	[29]
Dentine	18,600	0.31	[29]
Enamel	84,100	0.3	[29]
Bone	1370	0.3	[28]
Cement	4000	0.35	[30]
Composite	16,600	0.24	[29]
HAP insert	100,000	0.28	[31]

## Data Availability

The original contributions presented in the study are included in the article, further inquiries can be directed to the corresponding authors.
